# Endoscopic versus laparoscopic resection of gastric gastrointestinal stromal tumors: a multicenter study

**DOI:** 10.18632/oncotarget.13298

**Published:** 2016-11-11

**Authors:** Wei-Jie Dai, Gao Liu, Min Wang, Wen-Jie Liu, Wei Song, Xiao-Zhong Yang, Qi-Long Wang, Xiao-Yu Zhang, Zhi-Ning Fan

**Affiliations:** ^1^ Institute of Digestive Endoscopy and Medical Center for Digestive Diseases, The Second Affiliated Hospital of Nanjing Medical University, Nanjing, China; Department of Gastroenterology, Huai'an First People's Hospital, Nanjing Medical University, Huai’an, China; ^2^ Department of Gastrointestinal Surgery, Central Hospital of Enshi Autonomous Prefecture, Enshi Clinical College of Wuhan University, Enshi, Hubei, China; ^3^ Digestive Endoscopy Center, Jiangsu Province Hospital, The First Affiliated Hospital with Nanjing Medical University, Nanjing, China; ^4^ Department of Gastroenterology, Huai'an First People's Hospital, Nanjing Medical University, Huai’an, China; ^5^ Department of Clinical Oncology, Huai'an First People's Hospital, Nanjing Medical University, Huai’an, China; ^6^ Division of Gastrointestinal Surgery, Department of General Surgery, The Affiliated Huai'an Hospital of Xuzhou Medical University and The Second People's Hospital of Huai'an, Huai'an, Jiangsu, China; ^7^ Institute of Digestive Endoscopy and Medical Center for Digestive Diseases, the Second Affiliated Hospital of Nanjing Medical University; Digestive Endoscopy Center, Jiangsu Province Hospital, The First Affiliated Hospital with Nanjing Medical University, Nanjing, China

**Keywords:** minimally invasive, resection, gastric GISTs, endoscopic, laparoscopic

## Abstract

Despite endoscopic resection has been performed to treat gastric gastrointestinal stromal tumor (GISTs). However, the safety and long-term outcomes remains controversial. This study aims to compare the safety and surgical outcomes of endoscopic versus laparoscopic resection of gastric GISTs. A total of 335 patients that were pathologically confirmed with gastric GISTs (tumor size ≤ 3.5 cm) were surgically treated with endoscopic resection (endoscopic group) or laparoscopic resection (laparoscopic group) in three institutions from March 1, 2011 to October 1 2014. These demographics, tumor characteristics, and outcomes were retrospectively analyzed for identification of outcomes and feasibility of endoscopic or laparoscopic resection. Of 335 patients, 262 and 73 patients underwent endoscopic and laparoscopic resection, respectively. The average tumor size was 1.33±0.78 cm in the endoscopic group and 1.97±0.93 cm in the laparoscopic group. The average operating time was 62.40±36.94 min in the endoscopic group and 112.81±55.69 cm in the laparoscopic group. Days of realimentation was 2.76±1.67 in the endoscopic group and 4.89±2.03 in the laparoscopic group. The average cost was $ 3246.01±1017.61 in the endoscopic group and $ 4884.81±1339.51 in the laparoscopic group. There was no postoperative mortality. Endoscopic resection for gastric GISTs is safe and feasible in tumors ≤ 3.5 cm. Because endoscopic resection showed good results with lower operating time, realimentation days, length of hospital stay and mean total cost, it is a minimally invasive and safe alternative approach which can achieve fast recovery and satisfactory outcomes for appropriately selected patients with gastric GISTs.

## INTRODUCTION

Gastrointestinal stromal tumors (GISTs) are the most common mesenchymal tumor in the gastrointestinal tract [1, 2], the most common of which are KIT or PDGFRα (platelet-derived growth factor receptor alpha) activation mutations [3]. As a unique disease entity, it is estimated that the annual incidence of GISTs in the world is about 7 to 19 individuals per million [4–7]. The median age at onset was about 60 years old, with biologically distinct subsets in the pediatric age group [8, 9]. GIST is common in the stomach, followed by the intestine. However, considerable GISTs are found in the colon, esophagus and other parts of the peritoneal cavity [10, 11].

Normally, histological criteria show that malignancy tumors do not consistently exhibit aggressiveness. Alternatively, some tumors with typical “benign” characteristics cause metastasis. Tumor size and mitotic counts are recognized to assess prognosis. Using both indices, Fletcher and colleagues were able to classify patients with primary GISTs into four risk groups and to predict aggressive behavior. [12]. A model with a large number of patients with GISTs suggested that the anatomical location was the currently accepted risk model for local GISTs, and that the primary disease site, together with tumor size and mitotic count, provided a model of the risk of future relapse after local disease resection [10].

Localized GISTs treatment regimen including complete surgical resection. Lymph node dissection is not a standard practice, because tumor spread is usually blood-borne, rather than through the lymphatic system. If complete surgical resection with negative margins (R0 resection) is not the first attempt to achieve, can be done safely through repeated surgery, this option can be considered. Surgery is applicable to primary resectable GISTs, and is the only radical treatment. Although radical resection is performed in localized GISTs, about 40% of patients will relapse and eventually die from this disease [13, 14]. Therefore, surgical resection should be considered in carefully selected patients with limited progressive disease that is potentially easily resectable [15–17].

Recent studies have shown that endoscopic resection is safe and viable for patients with GISTs, even in tumors up to 5 cm in size [18]. In theory, endoscopic resection is a simple and feasible treatment for some tumors, but the risk of early tumor recurrence with incomplete resection is a major concern for the surgeon. In contrast, laparoscopic resection is widely accepted to remove the entire tumor from a technical and oncological point of view, leading to a histologically negative margin effect. However, as far as we know, although the previous case series reported the preliminary efficacy of endoscopic resection for the treatment of gastric GISTs, there have been few studies comparing the safety and surgical outcomes of endoscopic and laparoscopic resection of gastric GISTs. Therefore, we conducted the multicenter clinical study to evaluate the advantages and disadvantages of endoscopic resection and proven laparoscopic resection in the treatment of gastric GISTs.

## RESULTS

### Patients

**Figure 1:**
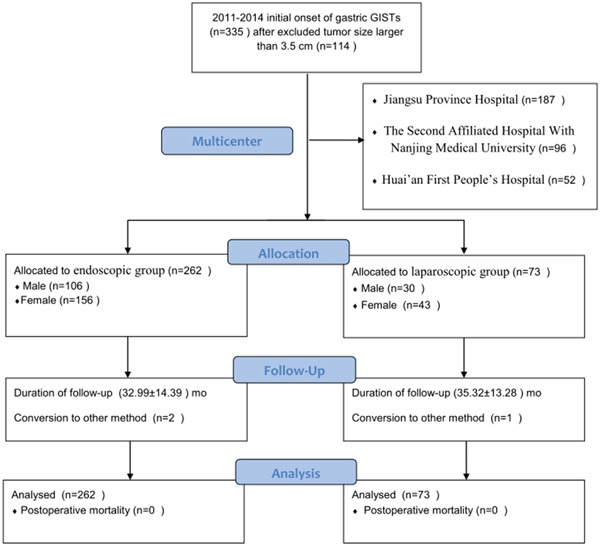
Flowchart of patients inclusion and exclusion

According to the patient inclusion and exclusion criteria, 335 patients were included into this study finally, with 262 patients underwent endoscopic resection (endoscopic resection group) and the other 73 patients underwent laparoscopic resection (laparoscopic resection group) (Figure 1). The clinical and pathologic characteristics of 335 patients from three hospitals are shown in Table 1. The location of the tumor included 189 cases in gastric fundus, 85 cases in gastric body, 39 cases in gastric cardia, and 14 cases in gastric antrum. The average age was 57.00 ± 10.32 years (range, 23 to 81 y) in the endoscopic resection group and 57.95 ± 11.89 years (range, 28 to 83 y) in the laparoscopic resection group. The mean GIST tumor size was 1.33 cm in the endoscopic resection group and 1.97 cm in the laparoscopic resection group. Approximately 79.1% of the patients were symptomatic and 21.0% of patients had more than five mitotic figures per fifty high power field (HPF). According to NIH risk classification, approximately 8.2% of patients had the risk from intermediate to high. There were 95.3% (241/253) and 97.6% (247/253) of the whole population CD-117 and CD-34 positive respectively. The size of GIST tumors in patients undergoing endoscopic and laparoscopic resection were in the range of 0.2 to 3.5 cm and 0.4 to 3.5 cm, respectively.

**Table 1:** Baseline characteristics of patients

		Endoscopic resection (n=262)	Laparoscopic resection (73)	*P* value
Age	Median (Range)	58 (23-81)	60 (28-83)	
	Mean±SD	57.00±10.32	57.95±11.89	0.540
Gender	Male (n=136)	106	30	1.000
	Female (n=199)	156	43	
Symptoms	Abdominal pain (n=193)	157	36	0.965
	Bleeding (n=16)	4	12	
	Others (n=56)	47	9	
	No symptoms (n=70)	54	16	
Combined with ulceration	Yes (n=18)	6	12	0.130
	No (n=313)	252	61	
Tumor location	Gastric fundus (189)	154	35	0.827
	Gastric body (85)	59	26	
	Gastric antrum (14)	7	21	
	Gastric cardia (39)	34	5	
Tumor size (cm)	Mean±SD	1.33±0.78	1.97±0.93	0.000
Mitotic rate (per 50 HPF)	≤5cm (83)	59	24	0.255
	5-10cm (20)	16	4	
	>10cm (2)	2	0	
CD34 positive	Yes (n=247)	183	64	0.653
	No (n=6)	4	2	
CD117 positive	Yes (n=241)	178	63	1.000
	No (n=12)	9	3	
NIH risk classification	Very low risk (0)	0	0	0.255
	Low risk (179)	124	55	
	Intermediate risk (14)	11	3	
	High risk (2)	2	0	

CD34, CD34 protein; CD117, CD117 protein;HPF, High-power field; NIH, National Institutes of Health; SD, standard deviation.

### Perioperative outcomes

The surgical outcomes and postoperative courses are summarized in Table 2. A total of 262 gastric GISTs patients underwent endoscopic gastrectomy. The average operative time was 62.40±36.94 minutes. There was no postoperative mortality. Two patients due to close adhesion of the tumor to the stomach wall, endoscopic resection was not successful, the final implementation of the laparoscopic resection.

**Table 2:** Surgical outcomes and postoperative courses of patients

		Endoscopic resection (n=262)	Laparoscopic resection (73)	*P* value
Duration of follow-up (months)	mean±SD (262:73)	32.99±14.39	35.32±13.28	0.196
Conversion to other method	Yes (3)	2	1	0.522
	No (319)	250	69	
Resection margin	R0 (33)	2	31	/
	R1 (0)	0	0	
	R2 (0)	0	0	
Complications	None (316)	245	71	0.505
	Major bleeding (2)	2	0	
	Perforation (3)	3	0	
	Infection (7)	6	1	
	Intestinal obstruction (0)	0	0	
	Other (5)	4	1	
Operating time (min)	Mean±SD (245:72)	62.40±36.94	112.81±55.69	0.000
Realimentation (days)	Mean±SD (218:56)	2.76±1.67	4.89±2.03	0.000
Length of hospital stay (day)	Mean±SD (259:73)	5.47±2.10	8.21±2.64	0.000
Mean cost ($)	Mean±SD (262:73)	3246.01±1017.61	4884.81±1339.51	0.000
Mean cost (Ɏ)	Mean±SD (262:73)	21960.53±6884.53	33027.37±9062.31	
Postoperative mortality	Yes (0)	0	0	/
	No (281)	219	62	
Recurrence	Yes (2)	2	0	1.000
	No (279)	217	62	

SD, standard deviation.

**Figure 2:**
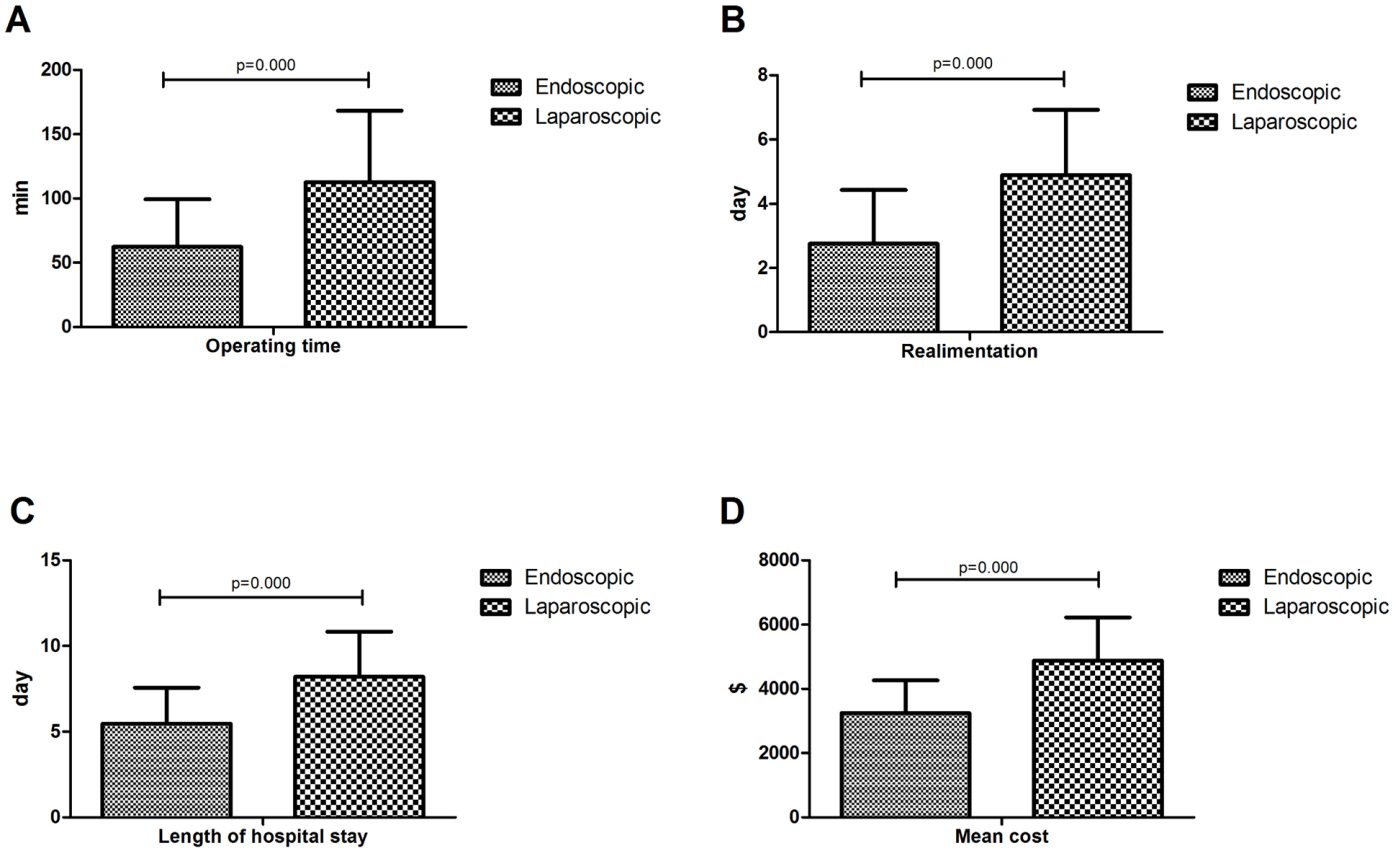
**A**. Operating time (min) of patients by treatment options; **B**. Realimentation (days) of patients by treatment options; **C**. Length of hospital stay (days) of patients by treatment options; **D**. Mean cost ($) of patients by treatment options.

The average of operative time in laparoscopic group was 112.81±55.69 minutes (Figure 2A). No lacrimal rupture or spillage occurred during laparoscopic surgery. Except one, all patients achieve negative surgical margins. The 52-year-old woman had a 3.5-centimeter GIST at the cardia. The patient eventually underwent open surgery and no residual tumor was found on the resected specimen. All patients undergoing laparoscopic surgery had no postoperative mortality. The mean length of postoperative realimentation were 2.76 and 34.89 days, respectively (Figure 2B).

In our current study, 335 consecutive hospitalized patients in three hospitals were successfully resected with a mean length of hospital stay of 5.47±2.10 day in the endoscopic resection group and 8.21±2.64 min in the laparoscopic resection group (Figure 2C), and the Mean cost ($) was limited to 3246.01±1017.61 in the endoscopic resection group and 4884.81±1339.51 in the laparoscopic resection group (Figure 2D).

### Tumor-related outcomes

The Tumor-related outcomes are summarized in Table 2. The whole follow-up period for all 335 patients from three hospitals was finally completed in June 2016. At a mean follow-up of 32.99±14.39 months in the endoscopic resection group and 35.32±13.28 months in the laparoscopic resection group, only two patients (0.72%) from the endoscopic resection group had a relapse, optimistically, these two patients did not find local recurrence. Eventually these two relapses were treated with imatinib and survived. No differences were observed in the conversion to other method rate (2/252 in the laparoscopic resection group vs 1/70 in the laparoscopic resection group, P = 0.522) and postoperative complications (15/260 in the laparoscopic resection group vs 2/73 in the laparoscopic resection group, P = 0.505) between these two groups.

## DISCUSSION

GISTs are the most common mesenchymal tumor in the gastrointestinal tract, the most common of which are KIT or PDGFRα activation mutations [3]. Sporadic GISTs, which account for more than 95% of cases, usually arise in middle-aged to older adults, with neither gender nor race predilection. No risk factors have yet been identified. For the reason, the distinction between malignant and benign GIST has historically been difficult to elusive. The malignant potential of a GIST is unpredictable because small tumors or tumors with an appropriate number of mitotic figures can be observed metastases or recurrences too. Gastrointestinal stromal tumors are typically solitary neoplasms, mainly originating in stomach (60%), small intestine (30%), rectum (5%), and esophagus (5%). By immunohistochemistry, most GISTs are positive for DOG1 (found on GISTs) and KIT (CD117), and often also for CD34. These stains are helpful in the differential diagnosis of morphologically similar intra-abdominal lesions. [13] Complete surgical excision is the treatment of choice and the only known curative therapy for primary localized, resectable GIST. However, approximately half of patients undergoing macroscopically complete surgical resection will experience disease recurrence within the following 5 years. [43] For decades pathologists have attempted to identify macroscopic or microscopic markers that could predict the risk of recurrence of localized GIST after surgery. The only features that have proved to be predictive of GIST behavior are tumor size and mitotic rate.

Some studies have shown that a safety margin of 1 to 2 cm includes a possible 5-mm micro-extension in GISTs leads to complete resection [19, 20] However, the controversy in the surgical margin is still under investigation. Because each GIST is now considered potentially malignant, all stromal tumors are recommended for surgical resection. Our results show that only 2 (0.72%) of the patients with tumors <3.5 cm have a high mitotic index consistent, further validate the above points. Compared with open surgery, laparoscopic resection of gastric stromal tumors has been shown to be feasible, safe, and has an excellent incidence of complications, [21, 22] endoscopic resection, a new technique for the treatment of gastric GIST, represents a less invasive alternative to surgical procedures. Endoscopic resection can be safely performed by experienced endoscopic specialists, and overall resection is preferable to local resection to accurately assess the adequacy of treatment. However, endoscopic resection of the gastric GISTs still has some problems. For example, the incidence of intraoperative bleeding, perforation and resection failure is high [23, 24]. In this study, the incidence of perforation and resection failure were 1.15% and 0.91% in endoscopic resection patients. Second, few studies have reported that endoscopic resection is suitable for GIST. Stromal tumors accurate preoperative diagnosis is mandatory for optimal therapy. Because the accuracy of endoscopic ultrasound needle biopsy has not yet been determined, so now is not considered a practical diagnostic method. Therefore, the safety of endoscopic resection in GISTs should be carefully studied. Our results suggest that when the tumor neointima grows well on the edge and there is a potential muscle layer under endoscopic ultrasonography, endoscopic resection can be used to the GISTs (less than 3.5 cm) and select the cases with high surgical risk, morbidity, or the need to preserve organ function. However, its applicability to oncology principles should be further evaluated by more patients.

Prognostic factors of recurrence have been investigated for R0 or R1 surgical patients, GIST tumor size, mitosis and tumor location is considered to be significant and independent prognostic factor [10, 12, 25]. In addition, rare tumor rupture has recently been identified as a prognostic factor [5, 26]. Macroinvasion may also be beneficial for patient risk stratification [26].

The recently reported findings have demonstrated a detailed survival rate of patients who underwent resection of locally excised gastric GIST. Although our study has shown satisfactory results in patients undergoing laparoscopic surgery for gastric GISTs, the retrospective analysis and the lack of comparative data on open or laparoscopic surgery as limitations of this clinical retrospective study. Thus, multi-center, prospective and comparative studies should be evaluated in endoscopic versus laparoscopic surgery for the different sizes of gastric stromal tumors.

In summary, laparoscopic resection may be a preferred alternative to small-size (<3.5 cm) tumors for locally resectable gastric GISTs. In addition, endoscopic resection showed more excellent results with lower operating time, realimentation days, length of hospital stay and mean total cost. Endoscopic resection is a minimally invasive treatment, and has the advantages of rapid recovery and satisfactory efficacy for gastric GISTs patients. Endoscopic resection could be considered for the treatment of gastric GISTs less than 3.5 cm in diameter. Further studies should be evaluated by more patients.

## MATERIALS AND METHODS

### Patients

In this study, the data were retrospectively and prospectively collected from patients who underwent endoscopic resection (endoscopic group) or laparoscopic resection (laparoscopic group) for gastric GISTs between March 1, 2011 and October 1 2014 in three hospitals (Jiangsu Province Hospital, Nanjing Medical University, Jiangsu, China; The Second Affiliated Hospital With Nanjing Medical University, Jiangsu, China; Huai’an First People's Hospital, Nanjing Medical University, Jiangsu, China). This study was conducted according to institutional ethics guidelines and was approved by the institutional review board in each institution. All procedures followed were in accordance with the ethical standards of the responsible committee on human experimentation and with the Helsinki Declaration of 1964 and later versions. Informed consent was obtained from all patients for being included in the study. Written informed consents were obtained from the patients for the publication of this report and any accompanying medical records.

The following patients were included in this study: (1) patients with tumor diameter no larger than 3.5 cm based on preoperative endoscopic ultrasonography (EUS) and/or abdominal computerized tomography (CT) examination; (2) patients who had not taken aspirin, warfarin, or other nonsteroidal anti-inflammatory drug for at least 1 week before the endoscopic resection; (3) patients who had normal complete blood count, prothrombin time, and thrombin time; “normal” means within the normal range; (4) patients with no other malignant tumors; (5) and patients who were pathologically diagnosed as having gastric GISTs preoperatively or postoperatively. Patients who refused surgical intervention were excluded. The surgical approaches were decided according to the tumor growth pattern, EUS findings, or patients’ preference.

### Pathological diagnosis

When histopathology revealed spindle, epithelioid, or mixed features by hematoxylin and eosin (H&E) staining, and when immunohistochemical analysis showed KIT (CD117) and/or CD34 positivity, patients were diagnosed with GIST. The histopathological features, cell shape, and number of mitoses per 50 HPF were obtained by examination of H&E-stained specimens. Mitoses were counted at the highest power, and mean values were used for the analysis after counting the fields twice. For patients who lacked pathological data, including immunohistochemistry, we histologically re-examined their surgical materials by the pathologist, when their paraffin blocks were available and usable.

Patients were classified using the NIHC and AFIPC [5, 10]. Since the NIH consensus criteria do not specify how to classify tumors with exactly 5 mitoses per 50 HPF or tumors that are exactly 2, 5, or 10 cm in size, we defined mitosis and tumor size in the NIH consensus criteria as follows: <5/50 or ≥5/50 HPF, and ≤10/50 or >10/50 HPF for mitosis, and <2 or ≥2 cm, ≤5 or >5 cm, and ≤10 or >10 cm for tumor size. In brief, for the NIHC criteria, we stratified patients into 4 risk groups: very low risk, low risk, intermediate risk, and high risk. Two pathologists who were blinded to the data reviewed all the specimens; if their initial diagnoses differed, the pathologists would reassess the specimens and discuss their findings to reach the consensus. Characteristics of the patients such as their age and gender, those of the operation including the operating time and conversion rate, the size and location of the tumor as well as postoperative complications were recorded.

### Surgical management

For each patient, treatments were allocated based upon patient will and clinicopathological characteristics, which were assessed by the Expert Team in each Hospital. These teams comprised gastroenterologists, endoscopists and general surgeons. Written informed consent was obtained from each patient prior to surgical treatment.

All patients received general anesthesia. The surgical procedures included endoscopic resection (endoscopic group) and laparoscopic resection (laparoscopic group). Endoscopic resection was mainly chosen for patients with tumors originating from the muscularis propria or tumors with intragastric type and clear boundaries to adjacent tissues and organs. The patients were sedated using intravenous midazolam, with monitoring of the heart rate, blood pressure, and oxygen saturations. Conscious sedation was maintained with additional injections of midazolam during the procedure. Grasping forceps, an insulated-tip knife (KD-610L, Olympus Optical Co. Ltd), a hook knife (KD-620LR, Olympus Optical Co. Ltd.), and a polypectomy snare tip were used for dissection of the tumor complete with normal mucosa from surrounding tissue after injection of saline, including epinephrine (1:100000) and diluted indigo carmine dye. An electrocautery snare was used for coagulation in some cases. Complete endoscopic resection of gastric GISTs is regarded as the absence of any remnant of tumor visible on endoscopy after tumor resection. All endoscopic resections were performed by skilled endoscopic specialists and surgeons. Laparoscopic surgery was performed in reverse Trendelenburg position. After insertion of a subumbilical trocar by a Veress approach, the other 3 ports were added under laparoscopic observation. All procedures were performed by three experienced surgeons in their own hospitals. The surgeon stood between or to the left of the patient's legs. The first assistant was to the right or the left of the patient's body, and the gastroscope was placed at the left of the patient's head. Four ports (three 5 mm in diameter and one 12 mm in diameter) were inserted into the upper left and upper right quadrants at 10 mm. A 30°laparoscope was introduced through a subumbilical port following carbon dioxide (CO_2_) insufflations of up to 13 mmHg. A drainage tube was placed in the abdominal cavity in case intraoperative complications occurred. All patients were routinely managed using a standardized postoperative protocol.

### Patients follow-up

Patients were scheduled for follow-up with abdominal CT scanning and/or esophagogastroduodenoscopy according to risk classification, every 6 months during the first year of follow-up and every 12 months thereafter in those with very low or low risk of tumor recurrence, and every 3 months in the first year of follow-up and every 6 months thereafter in intermediate-risk patients and every 3 months in high-risk patients. Follow-up was completed by either chart review or telephone interview in August 1 2016.

### Statistical analysis

Statistical analyses were performed using SPSS 21.0 (SPSS Inc., Chicago, IL, USA). The age of the patients, together with the operating time, tumor size and the length of hospital stay were presented as mean ± standard deviation (SD). The categorical data were presented as number and percentage. The different characteristics were compared between the groups using a Chi-square test or Fisher's exact test for categorical variables and using a Student's t-test for continuous variables. P < 0.05 was considered as statistically significant.
